# Machine Learning for Predicting Pulmonary Graft Dysfunction After Double-Lung Transplantation: A Single-Center Study Using Donor, Recipient, and Intraoperative Variables

**DOI:** 10.3389/ti.2025.14965

**Published:** 2025-10-22

**Authors:** Julien Fessler, Cédric Gouy-Pailler, Wenting Ma, Jerôme Devaquet, Jonathan Messika, Matthieu Glorion, Edouard Sage, Antoine Roux, Olivier Brugière, Alexandre Vallée, Marc Fischler, Morgan Le Guen, Matthieu Komorowski

**Affiliations:** ^1^ Department of Anesthesiology, Hôpital Foch, Suresnes, France; ^2^ Department of Anesthesiology, Weill Cornell Medicine, New York, NY, United States; ^3^ CEA, List, Université Paris-Saclay, Palaiseau, France; ^4^ Department of Intensive Care Medicine, Hôpital Foch, Suresnes, France; ^5^ Department of Thoracic Surgery, Hôpital Foch, Suresnes, France; ^6^ Université Versailles-Saint-Quentin-en-Yvelines, Versailles, France; ^7^ Department of Pneumology, Hôpital Foch, Suresnes, France; ^8^ Department of Epidemiology - Data - Biostatistics, Delegation of Clinical Research and Innovation, Hôpital Foch, Suresnes, France; ^9^ Intensive Care Unit, Charing Cross Hospital, London, United Kingdom; ^10^ Division of Anaesthetics, Pain Medicine, and Intensive Care, Department of Surgery and Cancer, Faculty of Medicine, Imperial College London, London, United Kingdom

**Keywords:** lung transplantation, ECMO, primary graft dysfunction, machine-learning, gradient-boosting

## Abstract

Grade 3 primary graft dysfunction at 72 h (PGD3-T72) is a severe complication following lung transplantation. We aimed to develop an intraoperative machine-learning tool to predict PGD3-T72. We retrospectively analyzed perioperative data from 477 patients who underwent double-lung transplantation at a single center between 2012 and 2019. Data were structured into nine chronological steps, and supervised machine-learning models (XGBoost and logistic regression) were trained to predict PGD3-T72, with hyperparameters optimized via grid search and cross-validation. PGD3-T72 occurred in 83 patients (17.3%). XGBoost outperformed logistic regression, achieving peak performance at second graft implantation with an AUROC of 0.84 IQR: 0.065, p < 0.001, with a sensitivity of 0.81 and a specificity of 0.68. The top predictors included extracorporeal membrane oxygenation (ECMO) use, blood lactate levels, PaO2/FiO2 ratio, and total lung capacity mismatch. Subgroup analyses confirmed robustness across ECMO and non-ECMO cohorts. PGD3-T72 can be reliably predicted intraoperatively, offering potential for early intervention.

## Graphical Abstract

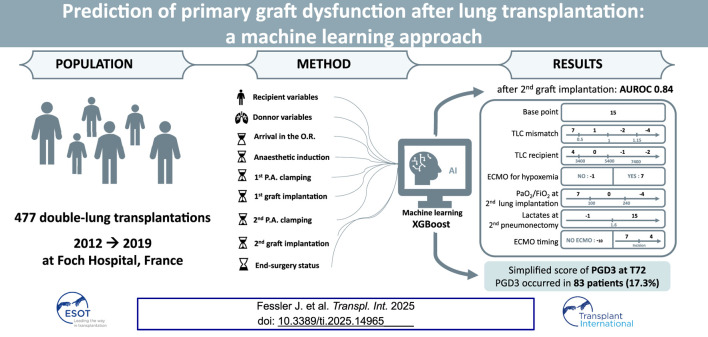

## Introduction

Following double-lung transplantations, grade 3 primary graft dysfunction at 72 h (PGD3-T72) is associated with increased risks of graft failure, bronchiolitis obliterans syndrome, and higher one-year mortality [1, 2]. Its incidence varies widely across centers, ranging from 3% to 25%, underscoring the need to reevaluate its risk factors while considering the evolving clinical practices. For instance, *ex vivo* lung perfusion has expanded the lung donor pool, extending the grafts’ ischemic times, with favorable outcomes [3, 4]. Likewise, tremendous strides have been made with the wider use of intraoperative extracorporeal membrane oxygenation (ECMO) [5] and its extension into the postoperative period [6]. Such dynamic changes in clinical practice, while beneficial for patients, can pose challenges in identifying risk factors for PGD3-T72 development using mathematical models. In fact, the complex interrelationships among these factors often complicate their integration into traditional linear regression models.

Emerging machine learning techniques are promising tools, offering the capacity to detect complex, non-linear relationships among numerous variables associated with PGD3-T72. These approaches have been successfully employed to predict outcomes in kidney [7], liver [8], and pediatric heart transplantation [9]. Yet, their application to lung transplantation remains limited [10–13], particularly in the perioperative setting. Recently, Michelson et al. compared four algorithms to predict PGD3-T72, using features selected via LASSO regression to guide graft selection [14]. Such tools hold potential for informing bedside decisions, though further development is needed to adapt intraoperative strategies dynamically as the surgical procedure progresses.

Building on this foundation, our study leverages a large, prospectively collected dataset with detailed, step-by-step intraoperative data from patients undergoing double-lung transplantation (DLT). We aimed to identify risk factors for PGD3-T72 and develop a simplified, clinically practical, risk scoring system.

## Materials and Methods

### Study Design

This retrospective analysis utilized a prospectively collected, single-center database, approved by the Ethics Committee of the French Society of Anesthesia and Critical Care (IRB No. 00010254–2019–019). All patients provided informed consent, and the data were anonymized in accordance with the International Society for Heart and Lung Transplantation (ISHLT) ethical guidelines. We included all DLT recipients at our center from January 2012 to December 2019, excluding those undergoing multiorgan transplantation, cardiopulmonary bypass, or retransplantation (if the index surgery was already collected and analyzed). Surgery involved two anterolateral thoracotomies with standardized anesthetic management, as previously described [15].

### Study Data and Variables

Anonymized data were prospectively collected in real-time during each surgery from patients’ electronic health records and stored using the FileMaker Pro database (FileMaker Company, Santa Clara, CA, USA). The transplantation process was divided into a nine-step analysis. Variables encompassing recipient and donor characteristics were entered into steps 1 and 2, respectively. Additionally, seven sequential surgical phases were entered into the analysis, step 3: arrival in the OR, step 4: post-anesthetic induction, step 5: first pulmonary artery clamping, step 6: first graft implantation, step 7: second pulmonary artery clamping, step 8: second graft implantation, and step 9: end-of-surgery status before ICU transfer (Table 1).

**TABLE 1:** Variables included in the model at each of the nine time points and their values.

Variables	PGD3 n = 83	No PGD3 n = 394	P
Step 1			
Age, years	41 [29–55]	40 [28–54]	0.98
Male gender	41 (49.4%)	198 (50.25%)	0.88
Weight, kg	59 [48–74]	54 [47–64]	0.03
Height, cm	165 [158–172]	166 [160–173]	0.75
Body mass index, kg.m^−2^	21 [18–25]	20 [18–22]	0.001
Total lung capacity, L	4.9 [3.2–6.3]	6 [4.9–7.5]	<0.001
Primary lung disease			
Cystic Fibrosis	34 (41%)	218 (55.3%)	0.017
COPD/Emphysema	9 (10.8%)	107 (27.2%)	0.001
Pulmonary Fibrosis	28 (33.7%)	39 (9.9%)	<0.001
Other	12 (14.5%)	30 (7.6%)	0.001
Retransplantation	2.4%	1.8%	0.70
Preoperative pulmonary hypertension*	32 (38.5%)	156 (39.6%)	0.86
Diabetes	20 (24.1%)	122 (31%)	0.21
Patent foramen ovale	7 (8.4%)	37 (9.3%)	0.65
Previous thoracic surgical procedure	19 (22.9%)	83 (21.1%)	0.71
Preoperative status			
Time on waiting list, days	15 [5–40]	18 [7–43]	0.22
Lung Allocation Score	38.6 [36.0–47.2]	36.7 [34.2–40.5]	<0.001
High emergency lung transplantation	13 (15.7%)	32 (8.1%)	0.03
Preoperative ICU	16 (19.3%)	43 (10.9%)	0.035
Preoperative mechanical ventilation	9 (10.8%)	9 (2.3%)	<0.001
Preoperative vasopressors	4 (4.8%)	10 (2.5%)	0.26
Prognostic Nutritional Index	45 [35–53]	45 [39–51]	0.86
Blood chemistry			
Hemoglobin, g/dL	11.9 [10.0–13.4]	11.9 [10.8–13.2]	0.48
Total bilirubin, µmol/L	1.8 [1.4–2.2]	1.6 [1.3–2]	0.08
Albumin, g/L	37 [28–41]	37 [31–42]	0.16
Creatinine, µmol/L	62 [46–82]	60 [49–73]	0.35
Creatinine GFR (MDRD ml/min)	119.7 [91.5–151.2]	118.7 [95.5–152.3]	0.48
Lymphocytes, G/L	1.7 [1.2–2.4]	1.5 [1.0–2.1]	0.07
Main treatment			
Preoperative antihypertensive drug	26 (31.3%)	125 (31.7%)	0.94
Preoperative antiplatelet therapy	10 (12%)	62 (15.7%)	0.39
Step 2			
Age, years	50 [42–59]	49 [37–61]	0.62
Male gender	51 (61.5%)	223 (56.6%)	0.42
Body mass index, kg.m^−2^	24.2 [21.1–26.2]	24.7 [22.1–27.7]	0.03
Estimated total lung capacity, L	6.5 [5.1–7.1]	6.4 [5.10–7.0]	0.41
Smoking history, pack-years	0 [0–19]	0 [0–12]	0.09
Bronchial aspirations			
Minimal, clear	39 (49.4%)	195 (52.1%)	<0.001
Moderate	8 (10.1%)	37 (9.9%)	0.006
Major, thick	31 (39.2%)	137 (36.6)	<0.001
Not Applicable	1 (1.2%)	5 (1.3%)	1
Chest X ray			
Normal	28 (33.7%)	132 (33.5%)	<0.001
Minimal	25 (30.1%)	91 (23.1%)	<0.001
Consolidation ≤1 lobe	16 (19.3%)	69 (17.5%)	<0.001
Consolidation >1 lobe	9 (10.8%)	85 (21.6%)	0.003
Not Applicable	5 (6%)	17 (4.3%)	0.03
PaO2/FiO2 ratio	357 [307–418]	362 [314–436]	0.18
Oto score	8 [6.5–11]	8 [6–10]	0.30
Length under mechanical ventilation, days	2 [1–3.5]	2 [1–3]	0.30
Maastricht III	0 [0–0]	0 [0–0]	0.30
Age mismatch	0.8 [0.6–1.1]	0.8 [0.6–1.2]	0.52
Gender mismatch	51 (61.5%)	247 (62.7%)	0.70
Total lung capacity mismatch	0.8 [0.5–1]	1 [0.8–1.2]	<0.001
Step 3			
Year of transplant	2016 [2015–2018]	2016 [2013–2018]	0.051
*Ex Vivo* lung perfusion	15 (18.1%)	87 (22.1%)	0.42
Preoperative plasmapheresis	36 (43.3%)	151 (38.3%)	0.39
Thoracic epidural analgesia	67 (80.7%)	349 (88.6%)	0.05
Step 4			
Hemoglobin concentration, g/dL	11.9 [10–13.4]	11.9 [10.8–13.2]	0.48
Blood lactate level, mmol/L	0.9 [0.7–1.35]	0.8 [0.6–1]	<0.001
Step 5			
Blood lactate level, mmol/L	1.2 [0.8–1.9]	1 [0.7–1.4]	0.003
Step 6			
Blood lactate level, mmol/L	2 [1.4–2.8]	1.5 [1.1–2.1]	<0.001
First lung ischemic time, min	282 [232–364]	284 [236–370]	0.96
Step 7			
Blood lactate level, mmol/L	2.3 [1.7–3.6]	1.5 [1.1–2.3]	<0.001
Step 8			
Blood lactate level, mmol/L	3 [2.2–4.8]	2.2 [1.7–3.2]	<0.001
Second lung ischemic time, min	432 [358–517]	412 [351–512]	0.36
PaO2/FiO2 ratio	156 [86–243]	242 [153–338]	<0.001
Step 9			
Graft lung reduction			0.005
None	56 (67.5%)	318 (80.7%)	<0.001
Wedge	6 (7.2%)	23 (5.8%)	0.02
Lobectomy	14 (16.8%)	39 (9.9%)	<0.001
Bilateral or >1 lobectomy	7 (8.4%)	14 (3.5%)	0.011
PaO2/FiO2 ratio	157 [94–236.5]	256 [172–360]	<0.001
Epinephrine use during surgery	15 (18.1%)	41 (10.4%)	0.05
Postoperative epinephrine requirement	16 (19.3%)	22 (5.6%)	<0.001
Norepinephrine infusion dose, µg/kg/min	0 [0–0.29]	0 [0–0]	0.025
Blood lactate level, mmol/L	3.3 [2.4–4.9]	2 [1.5–3.1]	<0.001
Estimated Blood Loss, L	1.4 [0.84–2.5]	1.0 [0.6–1.5]	<0.001
Packed Red Blood Cells, units	6 [4–10]	4 [3–6]	<0.001
Fresh-Frozen Plasma, units	6 [4–9]	4 [3–6]	<0.001
Platelet, Units	0 [0–1]	0 [0–0]	<0.001
Intraoperative fluid support, L	3 [2.5–4]	2.75 [2–3.5]	0.017
Inhaled nitric oxide dependence	14 (16.9%)	48 (12.2%)	0.25
Major intraoperative hemodynamic event	20 (24.1%)	13 (3.3%)	<0.001
Extubation in the operating room	3 (3.6%)	165 (41.9%)	<0.001

Results are expressed as n (%), or median [interquartile range].

Step 1: recipient variables, step 2: donor variables, step 3: arrival in the operating room, step 4: after anesthetic induction, step 5: first pulmonary artery clamping, step 6: first graft implantation, step 7: second pulmonary artery clamping, step 8: second graft implantation, and step 9: end-surgery status before transfer to the intensive care unit.

Age mismatch = recipient/donor.

TLC = total lung capacity is normalized on the height and gender [men = (height in cm x 7.992)-7.081; women =(height in cm x 6.602)-5.791].

Total lung capacity mismatch = recipient/donor (expressed as a continuous variable).

ECMO, extracorporeal membrane oxygenation.

COPD, chronic obstructive pulmonary disease.

iNO, inhaled nitric oxide;

Preoperative pulmonary hypertension*: number of patients with a mean pulmonary artery pressure >25 mmHg.

GFR: glomerular filtration rate.

Data regarding ECMO (time of insertion) are presented in Figure 2.

### Main Outcome

The incidence of PGD3-T72 was assessed per the 2016 ISHLT definition [16]. PGD3-T72 was graded by consensus by a board-certified panel including an intensivist, a pulmonologist, and an anesthesiologist. Patients on postoperative ECMO for hypoxemia were classified as grade 3. Predictive models were built for all nine steps, searching for the earliest high-discrimination step selected for clinical utility. We also compared the postoperative complications between patients who had PGD3-T72 and those who did not.

### Statistical Analyses

Authors followed the STROBE guidelines for observational studies.

All analyses were carried out in R (version 4.2.3). Normality of continuous variables was assessed using the Shapiro–Wilk test. Variables that conformed to a Gaussian distribution were described using mean and standard deviation and compared using the Student’s t-test. For non-normally distributed variables, we used median and interquartile range and performed comparisons using the Mann–Whitney U test. Categorical data are described as the number (percentage) and were compared using the Chi-squared test or Fisher’s exact test.

### Supervised Machine Learning Models

We employed supervised machine learning algorithms to predict PGD3-T72 in patients following double-lung transplantation (DLT). Supervised machine learning, a subset of artificial intelligence, involves training computer systems on labeled data to model the mathematical relationships between input features and outcomes [17–19]. In this study, we utilized the eXtreme Gradient Boosting (XGBoost) algorithm, which integrates multiple decision trees [19]. The weighted ensemble of these trees generates the final prediction [17–19]. For comparison, we benchmarked XGBoost against a baseline logistic regression (LR) model. To capture variation in clinical decision-making, particularly related to extracorporeal support, ECMO initiation timing was encoded as a categorical variable spanning six defined intraoperative periods (steps 4–9). While this does not directly model operator intent, it serves as a proxy for practice variation related to cannulation and intraoperative strategy.

### Data Preparation, Missing Data

No data transformation process was performed on the numerical variables. Categorical variables were one-hot encoded without any further preprocessing. Missing data was not imputed since XGBoost treats missing data as a specific modality. ECMO timing was encoded as a categorical variable using the following keys: 1: at second lung implantation; 2: at second pneumonectomy; 3: at first lung implantation; 4: at first pneumonectomy; 5: at induction of general anesthesia; and 6: preoperative ECMO.

### XGBoost Model Hyperparameter Tuning

We conducted hyperparameter tuning with the grid search approach and 5-fold cross-validation in 3 successive steps. First, we identified the optimal number of trees using a relatively high range of learning rates and standard values for the other hyperparameters (number of trees, maximum depth of each tree, regularization factor gamma, fraction of features by tree, minimum sum of instance weight needed in child tree, and subsampling rate). Then, we selected this number of trees, left the learning rate high, and conducted the grid search for all other parameters. Finally, in the third round, we fixed all hyperparameters and lowered the range of learning rates from 10E-5 to 10E-2.

The final chosen hyperparameters for the XGBoost model were: 50 trees, no early stopping, a maximum depth of 4 for each tree, a minimum sum of instance weight needed in child tree of one, a gamma of 0.75, and a learning rate of 10E-5. In addition to those conservative parameters chosen to prevent overfitting, only 40% of available columns were selected for tree construction in each round, and 95% of subjects were selected for tree construction (subsampling rate).

### Feature Selection and Final Model Training

Feature selection was performed using a recursive additive strategy within each of 500 randomly generated train/test splits. For each split, an XGBoost model was first trained on the full feature set to derive variable importance rankings (based on Gain), and then new models were retrained using incrementally larger subsets of top-ranked features (from 2 to 66) to evaluate area under the receiver operating characteristic curve (AUROC) on the corresponding test set.

While this approach involves out-of-sample testing on data not used for model training, feature selection was not nested within a formal cross-validation loop. A more rigorous nested cross-validation was deemed infeasible due to sample size constraints. As such, performance estimates may be modestly optimistic due to the potential for information leakage. However, to mitigate this risk, we repeated the full process 500 times, reporting median AUROC and interquartile ranges across iterations, and also included LR benchmarks using the same feature subsets.

### Model Performance Evaluation and Explanation Generation

We evaluated the performance of the XGBoost and LR models with their respective optimal number of features using standard metrics such as the AUROC, accuracy, sensitivity, specificity, positive predictive value, negative predictive value, precision, recall, and F1 score.

We used the SHapley Additive exPlanations (SHAP) methodology to generate post-hoc explanations for the model output. SHAP is based on game theory concepts and can be used to explain any machine learning model’s predictions by calculating each feature’s contribution to the prediction [20]. Specifically, we report the SHAP dependence plots, which represent the individual contribution of each selected feature to the outcome prediction.

All model performance metrics (e.g., AUROC, accuracy, sensitivity) were derived from the test set of each of the 500 random train/test splits. The final reported values are the median and interquartile ranges across these 500 out-of-sample estimates.

### Subgroup Analyses

Because ECMO has been previously highlighted as a major predictive factor of PGD3-T72 in our cohort [21], and to assess the robustness of our results in specific patient populations, we conducted subgroup and sensitivity analyses in patients who received ECMO at any time point (pre-operatively and/or perioperatively) patients who never received ECMO. We also performed a subgroup analysis on the cystic fibrosis population as they accounted for half of the cohort. Each subgroup analysis used the same hyperparameters as the full cohort and included 500 different models, each trained on different random train/test data splits.

### PGD3-T72 Simplified Risk Score

Using the top six features identified (from XGB) at surgical step 8, we trained an LR model to generate a clinically interpretable PGD3-T72 risk score. The model was developed as follows:

An LR model was fit using the training data subset of the full cohort (80% random split). We used the scorecard R package to convert the model’s regression coefficients into a simplified point-based risk score. Feature-specific cutoff values were determined using thresholds derived from SHAP dependence plots, which identify inflection points where changes in feature values significantly alter predicted risk. To validate the score, we performed 10-fold cross-validation using the full dataset to evaluate the discriminatory performance of the risk score. For clinical interpretability, the resulting score was grouped into six ascending risk bins, each corresponding to progressively higher observed rates of PGD3-T72. This binning strategy enhances bedside applicability and stratified decision-making.

## Results

**FIGURE 1:**
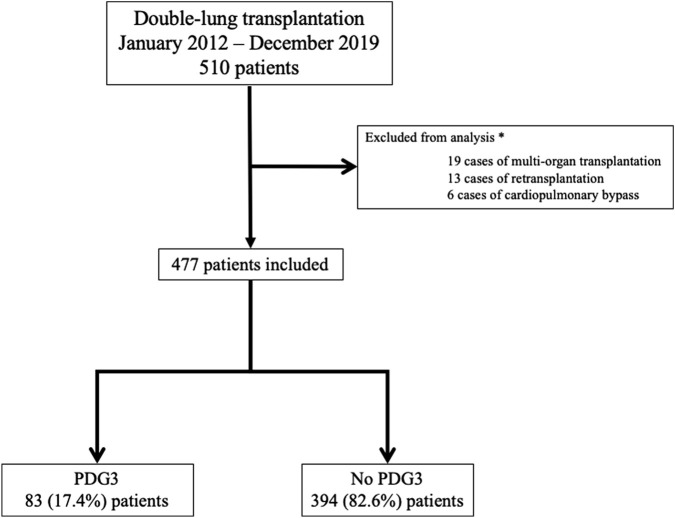
Flow chart. PGD3: Grade 3 pulmonary graft dysfunction at postoperative day 3. *: Some patients may have several reasons for exclusion.

The patient inclusion flowchart is depicted in Figure 1. Of the 510 patients who underwent double-lung transplantation (DLT) at our institution during the study period, 477 met the inclusion criteria and were analyzed (83 in the PGD3 group and 394 in the No PGD3 group).

Of these, in 455 cases the organs were sourced from brain-dead donors, while 22 cases involved donation after circulatory death.

Table 1 summarizes the data collected at each step. Our cohort reflected a large portion of cystic fibrosis patients (252, 52.7%) and no patients with primary pulmonary hypertension. Notably, 83 patients (17.3%) who developed a PGD3-T72 had a higher body mass index 21 [18–25] vs. 20 [18–22], p = 0.001, more elevated lactate at all time points (p < 0.001, expect p = 0.003 at step 5), but lower total lung capacity (TLC) 4.9 [3.2–6.3] vs. 6 [4.9–7.5], p = <0.001. Additionally, patients who met the criteria for the French High Emergency Lung Transplantation (HELT) program were overrepresented in the PGD3-T72 group (13 (15.7%) vs. 32 (8.1%) p = 0.03).

**FIGURE 2:**
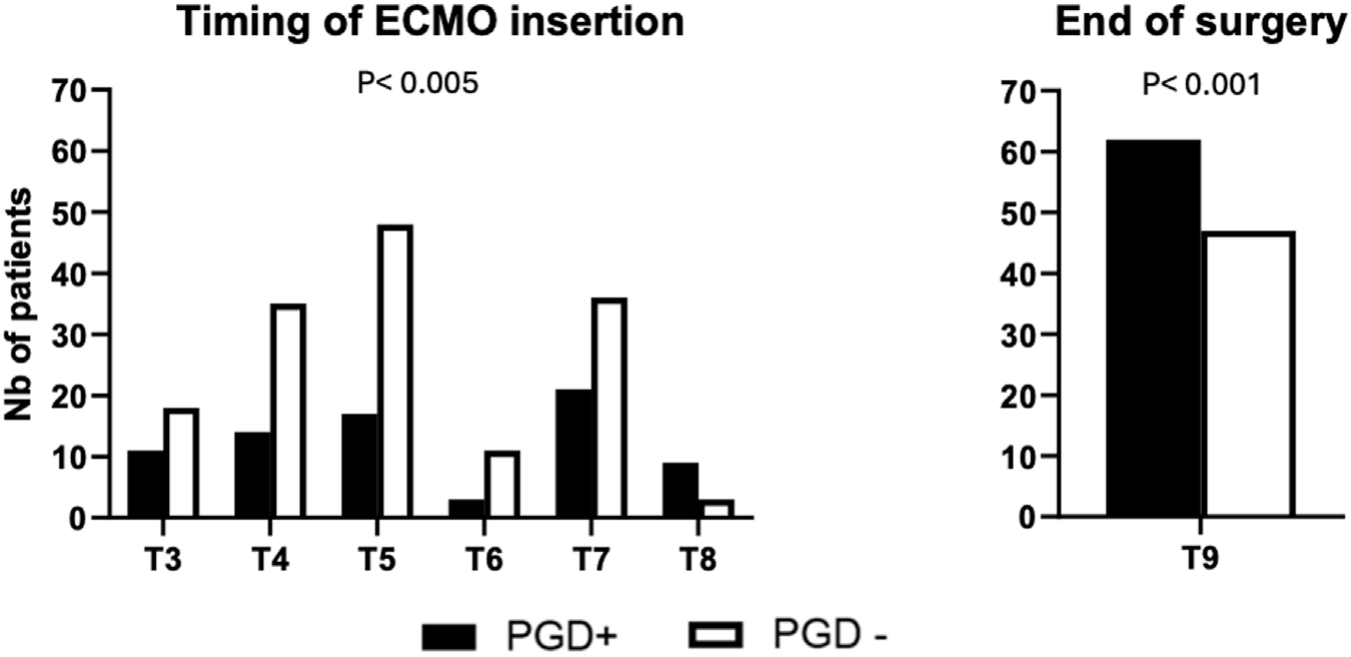
Time of ECMO cannulation T3 (arrival in the OR), T4 (after anesthetic induction), T5 (first pulmonary artery clamping), T6 (first graft implantation), T7 (second pulmonary artery clamping), T8 (second graft implantation), and T9 (end-surgery status before transfer to the intensive care unit). PGD+: Patients having a grade 3 primary graft dysfunction on postoperative day 3. PGD-: Patients not having a grade 3 primary graft dysfunction on postoperative day 3.

ECMO was not used in 251 patients, 7 (8.4%) in the PGD3+ group and 244 (61.2%) in the No PGD3 group (p < 0.001). On the other hand, 27 patients had ECMO in place upon arrival to the operating room: 11 (13.3%) in the PGD3+ group and 18 (4.6%) in the No PGD3 group (p = 0.003). The timing of ECMO cannulation, shown in Figure 2, differed significantly between groups (p = 0.005). Postoperatively, ECMO was continued in 62 (74.7%) patients in the PGD3+ group and 47 (11.9%) in the No PGD3 group (p < 0.001). Primary and secondary postoperative complications are detailed in Table 2.

**TABLE 2:** Primary and secondary postoperative complications.

Postoperative complications	PGD3 n = 83	Non PGD3 n = 394	*p*-value
Pulmonary complications			
Secondary intubation	10 (12.05%)	44 (11.17%)	0.818
Tracheotomy	39 (46.99%)	51 (12.94%)	<0.001
Total time under mechanical ventilation, days	10 (5–26.5)	0.5 (0–4)	<0.001
Secondary ECMO	18 (21.69%)	5 (1.27%)	<0.001
PGD3			
at T24	77 (92.77%)	90 (22.84%)	<0.001
at T48	80 (96.39%)	83 (21.07%)	<0.001
at T72	83 (100%)	0 (0%)	<0.001
Reoperation for bleeding	48 (57.83%	45 (11.42%)	<0.001
Postoperative transfusion			
Red blood cell packs, units	6 (2–15)	0 (0–1)	<0.001
Fresh frozen plasma, units	2 (0–7.5)	0 (0–0)	<0.001
Platelet, units	1 (0–2.5)	0 (0–0)	<0.001
Other complications			
Cerebrovascular accident	6 (7.22%)	6 (1.52%)	0.002
Renal replacement therapy	26 (31.33%)	8 (2.03%)	<0.001
Atrial fibrillation	26 (31.33%)	85 (21.57%)	0.056
Thromboembolic complication	37 (44.58%)	63 (15.99%)	<0.001
Lower limb ischemia	11 (13.25%)	5 (1.27%)	<0.001
Septic shock	40 (48.19%)	51 (12.94%)	<0.001
Length of stay and in-hospital mortality			
In the intensive care unit, days	16 (10–32)	5 (4–8.75)	<0.001
In the hospital, days	38 (24–73)	29 (24–39)	0.001
In-hospital mortality	24 (28.92%)	8 (2.03%)	<0.001

Values are n (%), or median (25th and 75th percentile). PGD3, grade 3 pulmonary graft dysfunction.

PGD3, primary graft dysfunction.

### Performance of the Predictive Models at all Analytical Steps

**FIGURE 3:**
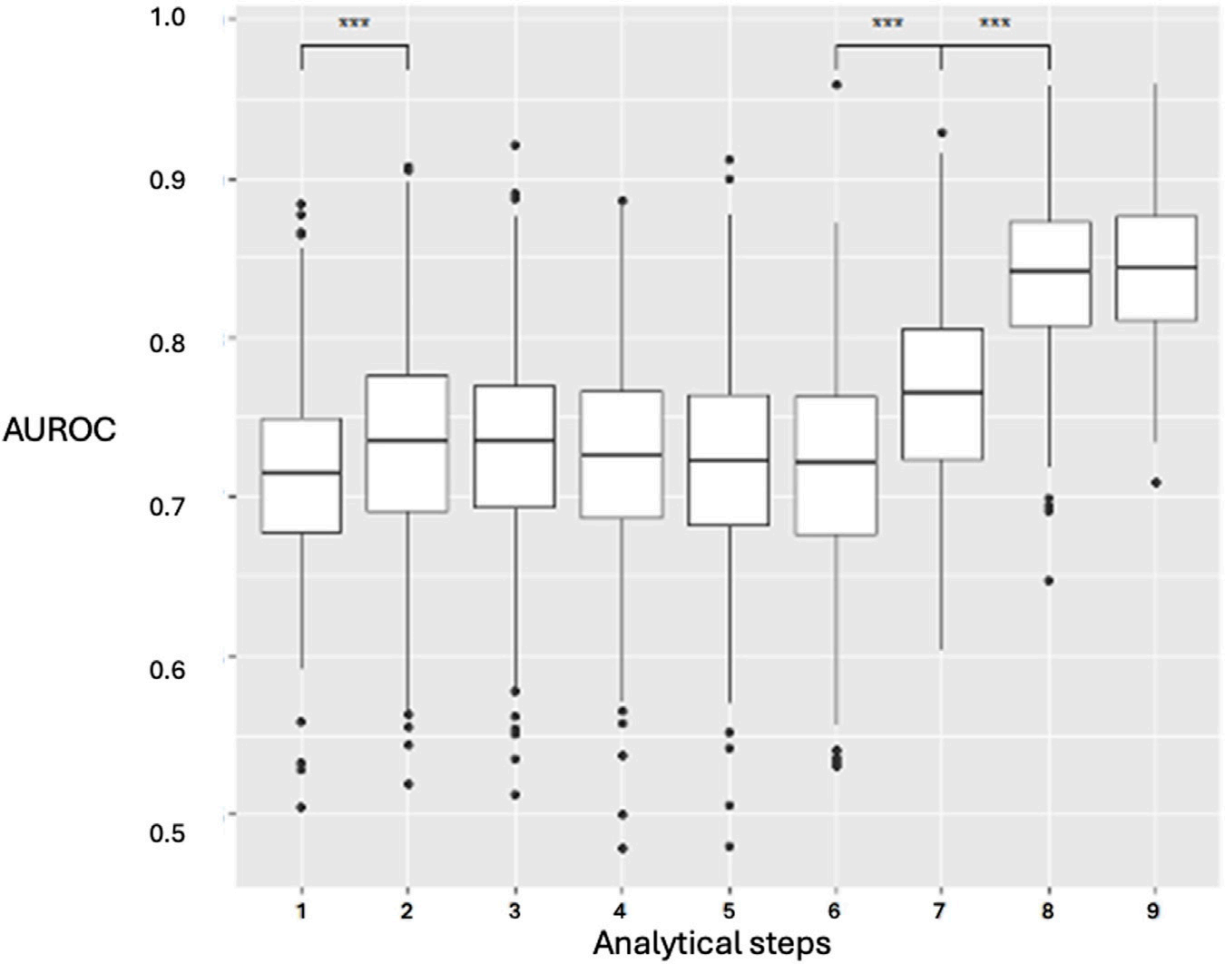
XGBoost prediction model for the nine clinical stages and analyzed steps. Data are presented as boxplots, where the limits of the boxes are defined by the first and third quartiles, and the whiskers extend to 1.5 times the interquartile range in each direction. AUROC: area under the receiver operating characteristic curve. Analytical steps are the following are the following: 1, recipient variables; 2, donor variables; 3, arrival in the OR; 4, anesthetic induction; 5, first pulmonary artery clamping; 6, first graft implantation; 7, second pulmonary artery clamping; 8, second graft implantation; 9, end-surgery status.

Incorporating an increasing number of features across the nine-step analysis enhanced the XGBoost model’s predictive performance (Figure 3). The AUROC was calculated in each fold, and the average cross-validated AUROC was 0.86 ± 0.01, indicating strong predictive accuracy and stability. The AUROC improved from step 1 to step 2, remained stable from step 2 to step 6, and then increased at step 7, peaking at step 8 (AUROC: 0.84, IQR: 0.065, p < 0.001, IQR: 0.065, p < 0.001). No further improvement was observed at step 9 (p = 0.19). Step 8 was selected as the earliest step with the highest AUROC. Confidence intervals were derived via bootstrapping, based on 500 iterations with different random train/test splits. Model performance using the top 6 features (XGBoost) and top 7 features (LR) is detailed in Supplementary Table 1.

### Performance of the Predictive Models at Surgical Step 8, Selection of Top Model Features

**FIGURE 4:**
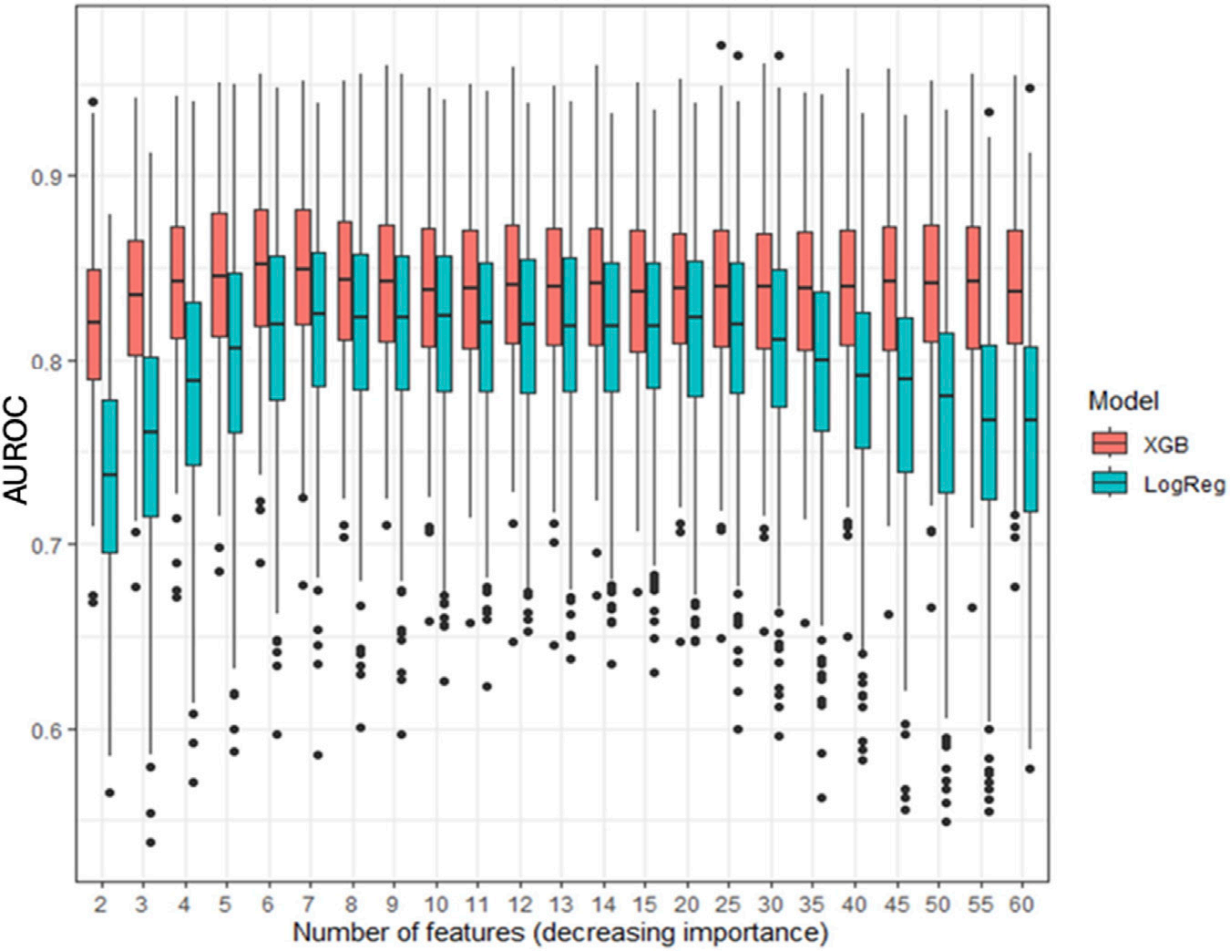
Evolution of the area under the curve for XGBoost (XGB) and logistic regression (LogReg) at surgical step 8, for an increasing number of features. Data are presented as boxplots, where the limits of the boxes are defined by the first and third quartiles, and the whiskers extend to 1.5 times the interquartile range in each direction.

**FIGURE 5:**
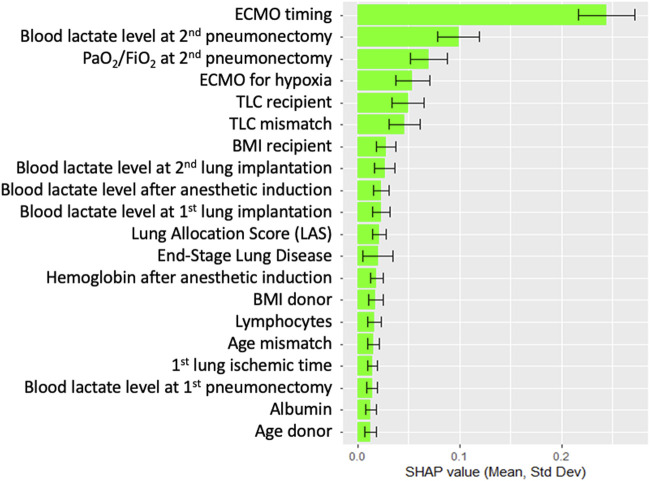
Top 20 features for XGBoost at surgical time step 8, ranked by order of decreasing importance. Data are presented as boxplots, where the limits of the boxes are defined by the first and third quartiles, and the whiskers extend to 1.5 times the interquartile range in each direction.

Figure 4 compares the AUROC for increasing features at step 8 using XGBoost and LR. XGBoost achieved the highest AUROC (0.84 ± 0.04) with 6 features, outperforming LR, which peaked at 7 features (AUROC 0.81 ± 0.05, sensitivity of 0.81, and specificity of 0.68). Figure 5 displays the top 20 features for XGBoost, ranked by decreasing importance. The relative importance (mean ± SD) of these top 20 features for the XGBoost model, based on the full cohort (N = 477) at surgical step 8, is reported. Comprehensive model performance metrics are provided in Supplementary Table 2.

### Model Interpretation: SHAP Dependence Plots for the Top 6 Features

**FIGURE 6:**
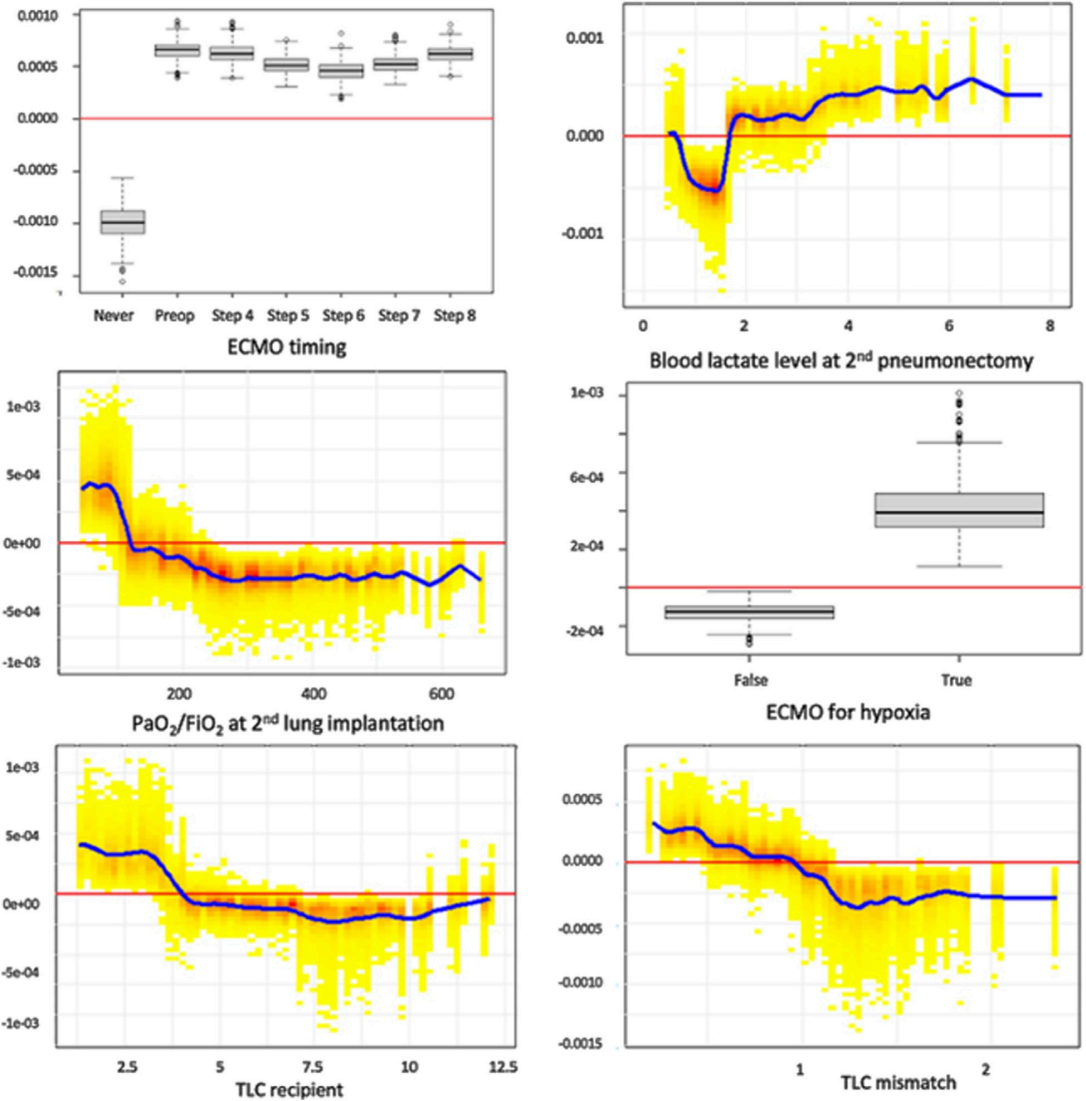
Individual SHAP dependence plots for the top 6 features of the XGBoost model. This analysis captures the non-linear relationship between feature value and the risk of PDG3, while accounting for all covariates in the model. Positive SHAP values reflect a positive association between the value of a feature (for example, high values of lactates at second pneumonectomy) and the risk of PGD3-T72, and *vice versa*. Blood lactate level is expressed in mmol/L. TLC recipients are expressed in liters. TLC: total lung capacity.

Figure 6 presents individual SHAP dependence plots for the top 6 features of the selected XGBoost model, illustrating the non-linear relationships between feature values and the outcome, such as TLC mismatch. As SHAP values reflect the marginal contribution of each feature within the model, we confirmed that ECMO use (at any time point) was independently linked to an elevated risk of PGD3-T72. Additional factors associated with increased PGD3-T72 risk included ECMO initiation for hypoxic indications, lactate levels exceeding 1.6 mmol/L after second pulmonary artery clamping, a PaO_2_/FiO_2_ ratio below 125 mmHg at first graft implantation, and a reduced recipient TLC.

### Subgroup Analyses

In the subgroup analysis, 251 patients underwent lung transplantation without ECMO. XGBoost achieved a median AUROC of 0.82 ± 0.09 at step 8 (Supplementary Figure 1; Supplementary Table 3). In a second subgroup analysis of 226 patients who underwent lung transplantation with ECMO at any time (preoperative and/or perioperative), the XGBoost analysis yielded an AUROC of 0.64 ± 0.04 (Supplementary Figure 2; Supplementary Table 4). Finally, the third subgroup analysis focused on the most represented end-stage lung disease, patients transplanted for cystic fibrosis (252 patients). The XGBoost analysis yielded an AUROC of 0.82 ± 0.04 (Supplementary Figure 3; Supplementary Table 5).

### Risk Score for PGD3

**FIGURE 7:**
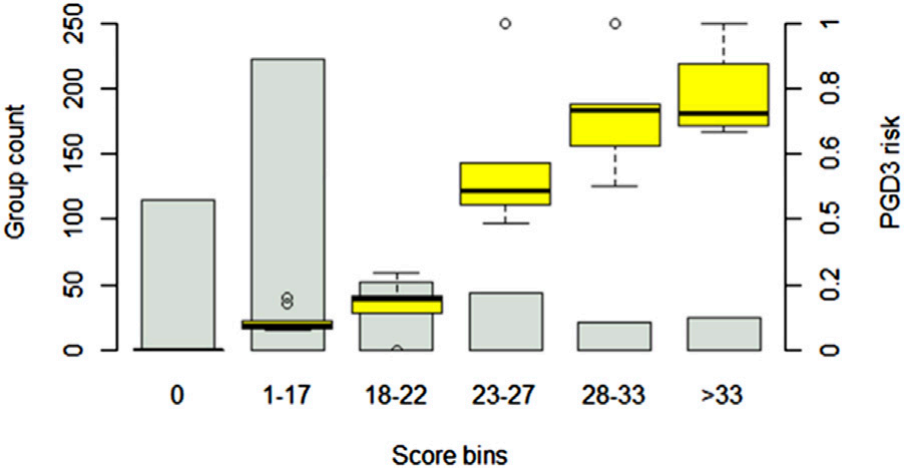
PGD3-T73 risk prediction score. The score is based on a logistic regression model using the top 6 features identified in the primary analysis. Patient scores are divided into six bins of increasing risk. The estimated risk of PGD3-T72 ranges from 0 (IQR: 0) (for a score of 0 or less) to 72% (IQR: 68%–87%) (for a score above 33 points). Confidence intervals are generated by testing the score on 500 random patient samples of varying sizes from the cohort, with resampling. The estimated risk of PGD3-T72 is represented as boxplots for each score bins.

The simplified risk score for PGD3 at T72 is presented in Table 3. The final score, calculated as the sum of the base points and each component, ranges from −7 to 62. Figure 7 illustrates the observed PGD3 rates across six distinct score bins. The estimated risk of PGD3 at T72 ranges from 0% (IQR: 0) for a score of 0 or below, to 72% (IQR: 68%–87%) for a score exceeding 33 points. The 10-fold cross-validated AUROC for the risk score is 0.86 ± 0.01.

**TABLE 3:** Simplified score of PGD3-T72.

Variable	Bin	Points
Base points		15
TLC mismatch	<0.5	7
TLC mismatch	[0.5, 1)	1
TLC mismatch	[1, 1.15)	−2
TLC mismatch	>1.15	−4
Recipient TLC	<3400	4
Recipient TLC	[3400, 5400)	0
Recipient TLC	[5400, 7400)	−1
Recipient TLC	>7400	−2
ECMO for hypoxic indication	No	−1
ECMO for hypoxic indication	Yes	7
PaO2/FiO2 ratio at step 8	<100	7
PaO2/FiO2 ratio at step 8	[100, 240)	0
PaO2/FiO2 ratio at step 8	>240	−4
Lactate concentration at step 7	<1.6	−1
Lactate concentration at step 7	>1.6	15
ECMO timing	No ECMO	−10
ECMO timing	Before surgery or at anesthetic induction	7
ECMO timing	Later than at anesthetic induction	4

The final score is the sum of the base points and of each component, and ranges from −7 to 62.

TLC, total lung capacity.

TLC mismatch, mismatch in total lung capacity between recipient and donor.

ECMO, extracorporeal membrane oxygenation.

## Discussion

Machine learning algorithms such as XGBoost offer a contemporary approach to clinical challenges [22]. Through automated variable selection, this method uncovered nonlinear relationships [23], adjusted for confounding factors, and delivered accurate, well-calibrated risk estimates. This study utilized such strengths of the XGBoost machine learning algorithm to predict primary graft dysfunction (PGD3) at 72 h (PGD3-T72) following lung transplantation. A distinctive feature of this research was the sequential development of predictive models at distinct stages of surgery, spanning from the assessment of recipient and donor characteristics to the transfer to the ICU. By progressively integrating intraoperative data, we determined that the highest predictive AUROC for PGD3-T72 was achieved after the second graft was implanted. We identified six key predictive features: recipient TLC and its mismatch with donor TLC, blood lactate levels (reflecting microcirculation), use of ECMO at any point (particularly for hypoxemia), and the PaO2/FiO2 ratio. These factors highlight the complex interplay of recipient characteristics, donor attributes, and intraoperative variables. Additionally, we developed a practical risk score based on these top six features to aid clinicians in assessing PGD3-T72 risk.

Importantly, the top predictors identified by our XGBoost model, including ECMO use, elevated lactate levels, impaired PaO_2_/FiO_2_ ratio, and donor-recipient total lung capacity mismatch, are consistent with previously published risk factors for primary graft dysfunction [5, 6, 24–26]. Our contribution lies in confirming these variables in a large, granular intraoperative dataset and integrating them into a unified, interpretable risk score with strong predictive performance. Since this scoring system can be implemented mid-surgery immediately after the second graft implantation, it can serve as an early prediction tool that provides clinicians with critical prognostic information, potentially allowing for timely adjustments in intraoperative or immediate postoperative management.

While our findings align with prior studies on PGD3-T72 risk factors, it also revealed novel associations, likely due to variations in institutional practices, evolving definitions of PGD3-T72, graft selection criteria, and intraoperative management [27–29]. In our study cohort, early predictors of PGD included elevated blood lactate at step 7, the PaO2/FiO2 ratio at step 8, and the use of ECMO for hypoxemia. These findings suggest that the pathophysiological mechanism driving the development of PGD likely begins at the stage of initial graft-host interaction, consistent with studies linking biomarker emergence to second graft implantation [24, 25, 30]. Additionally, it is worth noting that blood lactate was particularly predictive in patients who did not require ECMO, possibly underscoring the importance of maintaining adequate microcirculation during surgery.

Consistent with findings from a previous large retrospective cohort study [5], ECMO use was associated with increased incidence of PGD3-T72, regardless of timing. To further investigate the role of ECMO and its impact on model performance, we conducted subgroup analyses stratified by ECMO exposure. In the subgroup of patients who did not receive ECMO, the model achieved strong discriminatory performance (AUROC 0.82), with early intraoperative features, such as elevated lactate and low PaO_2_/FiO_2_ ratios after anesthetic induction, emerging as key predictors. These findings support the notion that early physiologic deterioration may represent a critical window for intervention, possibly advocating for a lower threshold for ECMO initiation to maintain cellular oxygen delivery in at-risk patients.

In contrast, in patients who received ECMO at any time (preoperative or intraoperative), the model’s performance was substantially reduced (AUROC ∼0.64). This diminished accuracy likely reflects the greater clinical heterogeneity in this subgroup, including variation in ECMO indications, timing of ECMO initiation, and preexisting severity of illness. In this context, the model may be confounded by complex decision-making patterns. Importantly, ECMO initiated specifically for hypoxemia (PaO_2_/FiO_2_ < 100 mmHg) remained a strong risk factor for PGD3, often occurring after second graft reperfusion, suggesting it may serve as an early clinical surrogate for emerging graft dysfunction.

Taken together, these findings indicate that the current risk score is best suited for use in non-ECMO patients or prior to ECMO initiation. In ECMO-supported patients, its interpretability and predictive power are more limited, and dedicated models tailored to this subgroup may be needed in future work [31, 32].

Another notable discovery is that recipient TLC emerged as a significant risk factor, independent of the type of end-stage lung disease. This may be attributed to the challenging surgical manipulation of severely retracted lungs in pulmonary fibrosis patients or the compromised nutritional status of cystic fibrosis patients [33]. However, TLC was not normalized to patient height in this analysis. Further research is needed to explore these specific patient groups, particularly to identify restrictive subpopulations with elevated chest wall elastance and to develop strategies for accelerating postoperative recovery of chest wall compliance [34].

In line with Tague et al., we found an optimal donor-recipient TLC ratio of 1.2–1.6, which prompted a practice shift following their publication, post-dating this cohort [26]. Such nonlinear relationships, obscured in traditional LR, underscore the value of machine learning.

Michelson et al. introduced a tool to support preoperative graft selection [14]. Our simplified score demonstrates superior discriminatory power, likely due to the inclusion of intraoperative factors affecting outcomes. Consequently, it serves as an effective instrument at the end of surgery for refining early postoperative approaches. Future studies could build on this foundation, developing tools with even greater AUROC values at later time points to optimize ICU postoperative care.

A key strength of this study lies in the detailed granularity of intraoperative data within our database, notably the comprehensive dataset organized around nine surgical steps, with systematic patient assessments at these specific time points. This structure enabled standardized data collection and its alignment with critical clinical moments. Another advantage is the use of a gradient boosting method, which, unlike LR, accommodates missing data without imputation, captures non-linear relationships, and delivers superior discrimination and calibration performance. Additionally, the application of state-of-the-art SHAP analysis provided an in-depth evaluation of how model features influence the risk of PGD3-T72, including the identification of clinically meaningful thresholds. Finally, we developed a simplified risk prediction score that avoids reliance on institution-specific variables, providing a practical tool for any transplantation center to assess PGD3-T72 risk effectively.

Our cohort predominantly featured cystic fibrosis patients, with primary pulmonary hypertension underrepresented due to recruitment patterns. While comprehensive, our dataset lacks variables such as immunologic compatibility and frailty. Unlike other studies, we prioritized early predictive factors to enable rapid clinical responses as primary graft dysfunction mechanisms emerge. Transfusion and fluid balance, introduced at step 9, did not enhance model performance [35, 36]. The repeated inclusion of ECMO-related variables, though unconventional in linear models, improved AUROC and was validated by supplementary analysis. A potential limitation of our study is the inability to explicitly account for variability in intraoperative decision-making, including differences in surgical technique, ECMO cannulation strategy, or operator-specific thresholds for intervention. Although our single-center setting with standardized surgical protocols helps mitigate this variability, some residual confounding is likely. Our model partially addresses this by encoding ECMO timing as a categorical feature, which may act as a surrogate for certain intraoperative choices. Nonetheless, future multi-center studies with access to surgeon- or institution-level metadata could benefit from hierarchical modeling frameworks to isolate operator-driven variability and better understand its impact on model generalizability. Another limitation is that, aside from LR, we did not evaluate a broader range of machine learning algorithms. While many supervised methods (e.g., random forests, support vector machines, deep neural networks) could potentially be applied, we selected XGBoost due to its strong empirical performance on structured data, built-in handling of missing values, and compatibility with SHAP-based interpretability. These characteristics make it well suited for real-time intraoperative applications. Future studies could compare alternative modeling strategies, including ensemble or hybrid architectures, to optimize performance and generalizability.

A key limitation of this study is the moderate sample size (n = 477), which may increase the risk of overfitting. To address this, we employed conservative hyperparameter settings and repeated random split validation, but future studies with larger multicenter cohorts are essential for external validation and generalizability.

Finally, an important limitation of this study is the absence of external validation. Despite outreach to several international centers through the ISHLT network, no collaborating institution was able to provide a dataset with comparable intraoperative granularity, particularly for stepwise modeling around second graft implantation. As a result, the model’s performance has only been demonstrated within a single center, and its generalizability to other clinical environments remains untested. Given known variability in transplant practices (including graft selection, ECMO initiation strategies, anesthetic techniques, and changing indications such as the increasing prevalence of pulmonary fibrosis), model performance may differ across settings. Thus, this model should be viewed as hypothesis-generating. We strongly advocate for prospective, multicenter cohort studies to validate perioperative machine learning models in diverse clinical contexts. To support reproducibility and facilitate such efforts, our full codebase has been made publicly available.

After validation of such models by a multicentric prospective study, the score could be implemented in a simple application or to the electronic record to alert clinicians on the possible risk of PGD3-T72. Therefore, it would suggest discussing within a preventive strategy. Furthermore, it could help to build future studies on prophylactic strategies to reduce PGD3-T72.

In conclusion, gradient boosting effectively predicted PGD3-T72 with an AUROC of 84% immediately after second graft implantation using routine intraoperative data. Further studies are needed to solidify machine learning’s role in primary graft dysfunction prediction and clinical practice. This tool could identify high-risk patients, enabling aggressive preventive measures to improve outcomes [37].

## Data Availability

Data are not publicly available due to privacy concerns. Requests to access the datasets should be directed to juf4007@med.cornell.edu.

## Supplementary Material

SUPPLEMENTARY IMAGES 1-2 Subgroup analysis of the 251 patients who underwent lung transplantation without requiring ECMO at any time. Panel **A**: AUROC for an increasing number of features at surgical step 8 with XGBoost. Panel B: top 20 features for XGBoost, ranked by order of decreasing SHAP importance. In panel **A**, data are presented as boxplots. In panel **B**, the data are presented as mean and standard deviation. Confidence intervals are generated by bootstrapping with N = 500 models, each with a different random train/test split, with resampling. In panel **C**: SHAP dependence plots for the top features. AUROC, area under the receiver operating characteristic curve; LogReg, logistic regression; XGB, eXtreme Gradient Boosting.

SUPPLEMENTARY IMAGES 3-4 Subgroup analysis of the 226 patients who underwent lung transplantation with ECMO at any time (preoperative and/or perioperative). Panel **A**: AUROC for an increasing number of features at surgical step 8 with XGBoost. Panel **B**: top 20 features for XGBoost, ranked by order of decreasing SHAP importance. In panel A, data are presented as boxplots. In panel **B**, the data are presented as mean and standard deviation. Confidence intervals are generated by bootstrapping with N = 500 models, each with a different random train/test split, with resampling. In panel **C**: SHAP dependence plots for the top features. AUROC, area under the receiver operating characteristic curve; LogReg, logistic regression; XGB, eXtreme Gradient Boosting.

SUPPLEMENTARY IMAGES 5-6 Subgroup analysis of the 252 patients who underwent lung transplantation for cystic fibrosis. Panel **A**: AUROC for an increasing number of features at surgical step 8 with XGBoost. Panel **B**: top 20 features for XGBoost, ranked by order of decreasing SHAP importance. In panel A, data are presented as boxplots. In panel **C**: SHAP dependence plots for the top features. In panel **B**, the data are presented as mean and standard deviation. Confidence intervals are generated by bootstrapping with N = 500 models, each with a different random train/test split, with resampling. AUROC, area under the receiver operating characteristic curve; LogReg, logistic regression; XGB, eXtreme Gradient Boosting.
