# Supplementary figures and images for: Machine Learning for Predicting Pulmonary Graft Dysfunction After Double-Lung Transplantation: A Single-Center Study Using Donor, Recipient, and Intraoperative Variables
Source: Transpl Int. 2025 Oct 22;38:14965. doi: 10.3389/ti.2025.14965 (PMC12593525; doi:10.3389/ti.2025.14965)

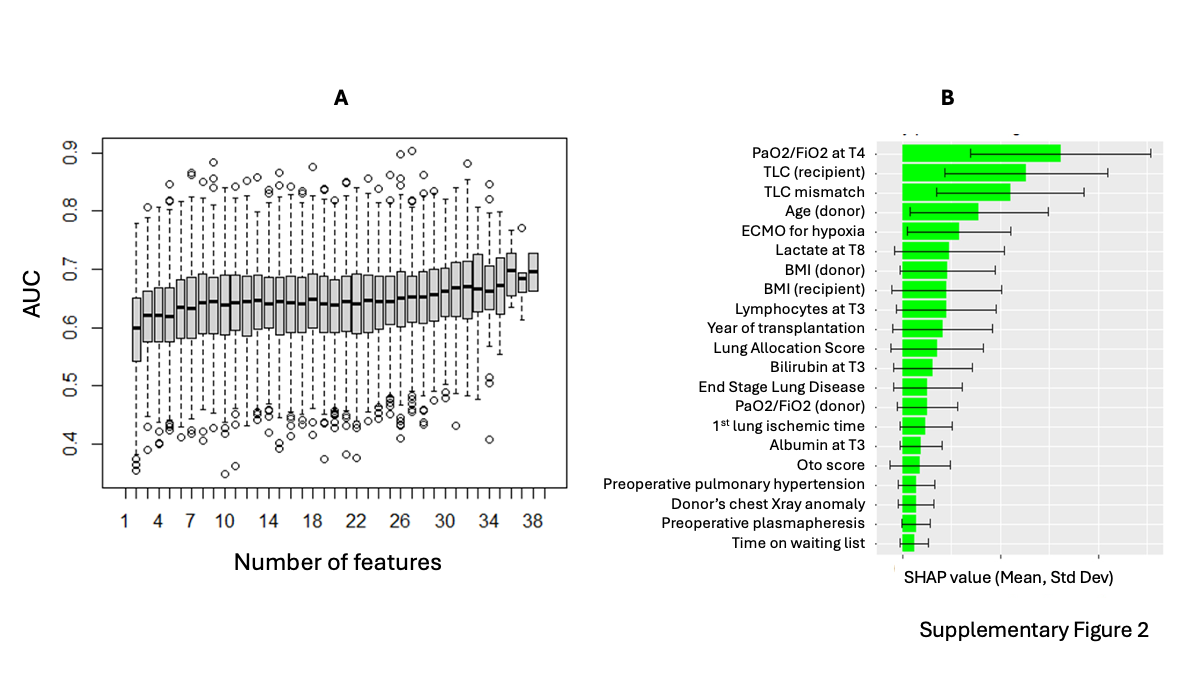

Supplement: Supplementary file 1 [file Image3.tiff]

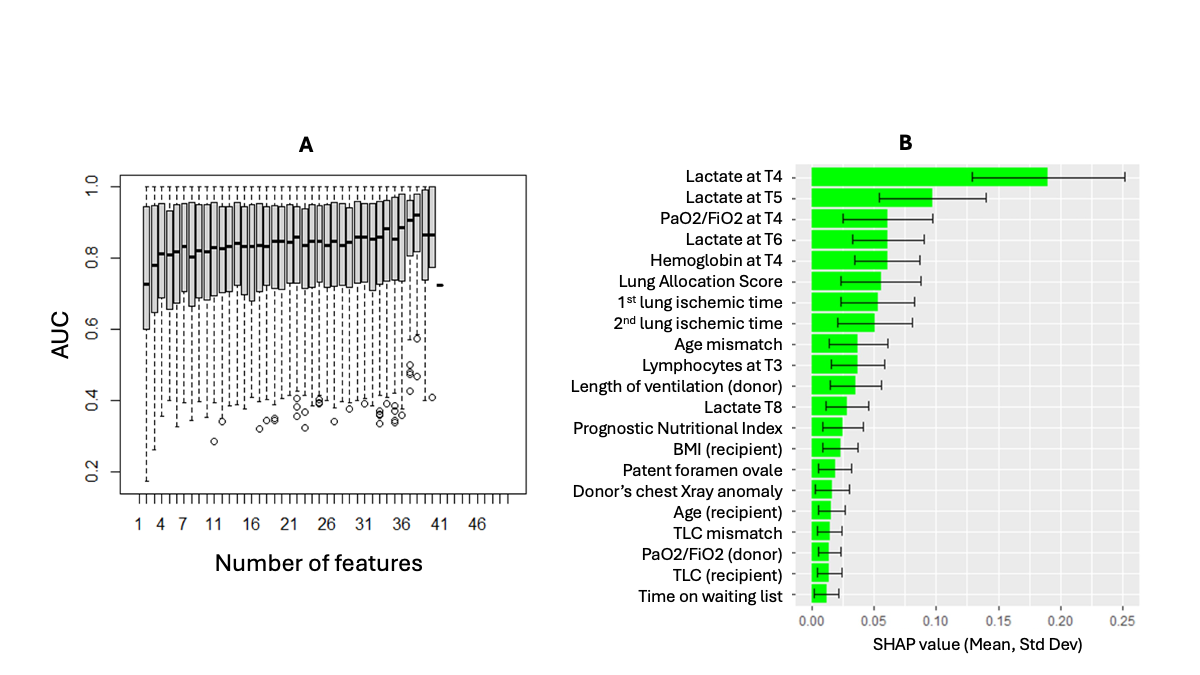

Supplement: Supplementary file 2 [file Image1.tiff]

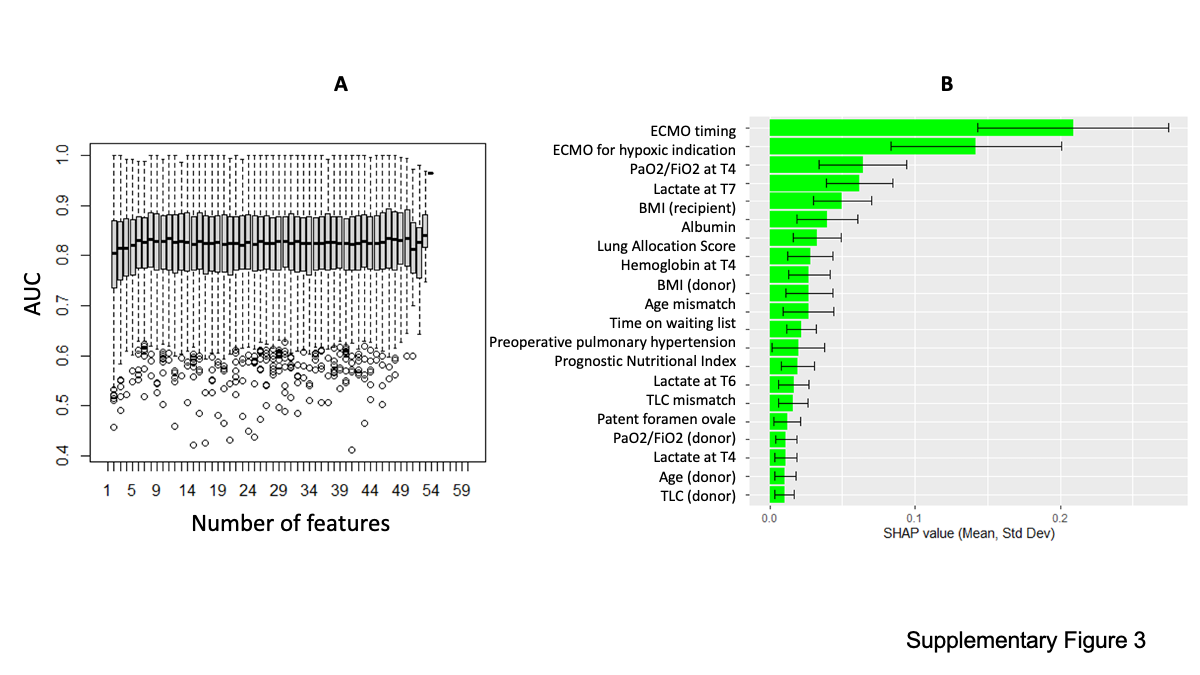

Supplement: Supplementary file 4 [file Image5.tiff]

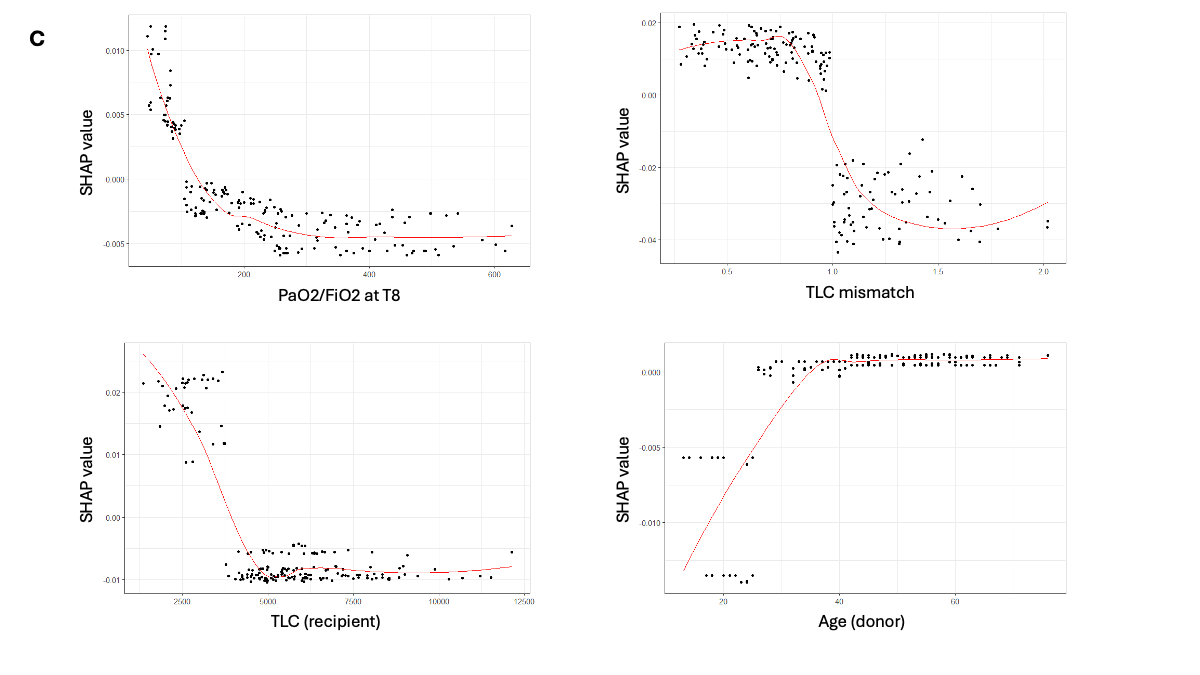

Supplement: Supplementary file 9 [file Image6.tiff]

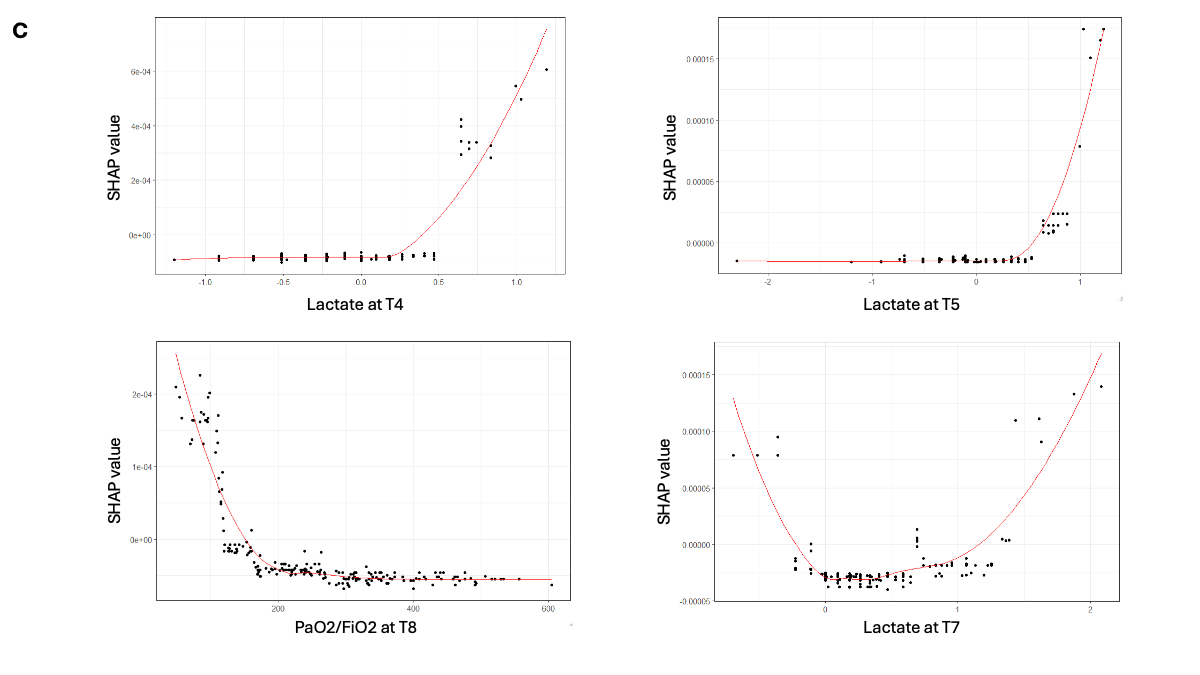

Supplement: Supplementary file 10 [file Image2.tiff]
