# Supplementary material for: Machine Learning for Predicting Pulmonary Graft Dysfunction After Double-Lung Transplantation: A Single-Center Study Using Donor, Recipient, and Intraoperative Variables
Source: Transpl Int. 2025 Oct 22;38:14965. doi: 10.3389/ti.2025.14965 (PMC12593525; doi:10.3389/ti.2025.14965)
Supplement: Supplementary file 3 [file Table1.docx]

**Supplementary Table 1**. Model performance with top 6 features (XGBoost - XGB) and top 7 features (Logistic regression - LR)

|  |  | **AUC** | **Accuracy** | **Sensitivity** | **Specificity** | **PPV** | **NPV** | **Precision** | **Recall** | **F1** |
| --- | --- | --- | --- | --- | --- | --- | --- | --- | --- | --- |
| XGB | Mean | **0.846** | 0.787 | 0.811 | **0.680** | **0.923** | 0.432 | **0.923** | 0.811 | 0.862 |
|  | SD | 0.043 | 0.041 | 0.050 | 0.120 | 0.032 | 0.101 | 0.032 | 0.050 | 0.028 |
| LR | Mean | 0.813 | **0.838** | **0.950** | 0.308 | 0.868 | **0.569** | 0.868 | **0.950** | **0.906** |
|  | SD | 0.055 | 0.033 | 0.026 | 0.109 | 0.034 | 0.170 | 0.034 | 0.026 | 0.020 |

Confidence intervals are generated by bootstrapping with N=500 models, each with different random train/test split, with resampling. Results are presented as mean and standard deviation (SD). AUC, area under the curve; PPV, positive predictive value; NPV, negative predictive value; F1, F1 score.
