# Supplementary material for: Machine Learning for Predicting Pulmonary Graft Dysfunction After Double-Lung Transplantation: A Single-Center Study Using Donor, Recipient, and Intraoperative Variables
Source: Transpl Int. 2025 Oct 22;38:14965. doi: 10.3389/ti.2025.14965 (PMC12593525; doi:10.3389/ti.2025.14965)
Supplement: Supplementary file 5 [file Table2.docx]

**Table S2**. Relative feature importance (mean, SD) of the top 20 features for the XGBoost model on the whole patient cohort (N=477), at surgical time step 8

| **Feature** |  |  |
| --- | --- | --- |
| ECMO timing | 0.244 ± 0.027 |  |
| Blood lactate level at 2^nd^ pneumonectomy | 0.099 ± 0.021 |  |
| PaO2/FiO2 at 2^nd^ lung implantation | 0.070 ± 0.018 |  |
| ECMO for hypoxia | 0.054 ± 0.017 |  |
| TLC recipient | 0.050 ± 0.016 |  |
| TLC mismatch | 0.046 ± 0.015 |  |
| BMI recipient | 0.028 ± 0.009 |  |
| Blood lactate level 2^nd^ lung implantation | 0.027 ± 0.010 |  |
| Blood lactate level after anesthetic induction | 0.023 ± 0.007 |  |
| Blood lactate level at 1^st^ lung implantation | 0.023 ± 0.008 |  |
| Lung Allocation Score | 0.021 ± 0.007 |  |
| End-Stage Lung Disease | 0.020 ± 0.015 |  |
| Hemoglobin after anesthetic induction | 0.019 ± 0.006 |  |
| BMI donor | 0.018 ± 0.007 |  |
| Lymphocytes | 0.017 ± 0.006 |  |
| Age mismatch | | 0.015 ± 0.005 |
| First lung ischemic time | 0.015 ± 0.004 |  |
| Blood lactate level at 1^st^ pneumonectomy | 0.015 ± 0.005 |  |
| Albumin | 0.013 ± 0.005 |  |
| Donor’s age | 0.013 ± 0.006 |  |

Confidence intervals are generated by bootstrapping with N=500 models, each with a different random train/test split, with resampling

Results are presented as mean ± standard deviation

BMI, body mass index; TLC, total lung capacity; ECMO, Extracorporeal membrane oxygenation
