# Supplementary material for: Machine Learning for Predicting Pulmonary Graft Dysfunction After Double-Lung Transplantation: A Single-Center Study Using Donor, Recipient, and Intraoperative Variables
Source: Transpl Int. 2025 Oct 22;38:14965. doi: 10.3389/ti.2025.14965 (PMC12593525; doi:10.3389/ti.2025.14965)
Supplement: Supplementary file 6 [file Table5.docx]

**Supplementary Table 5.** Relative feature important in subgroup analysis 3: Cystic fibrosis patients (N=252)

| **Feature** |  |
| --- | --- |
| ECMO timing | 0.208 ± 0.065 |
| ECMO for hypoxic indication | 0.142 ± 0.058 |
| Initial PaO2/FiO2 | 0.064 ± 0.030 |
| Lactates 2^nd^ pneumonectomy | 0.061 ± 0.022 |
| Body mass index of the recipient | 0.049 ± 0.020 |
| Albumin | 0.039 ± 0.021 |
| Lung Allocation Score | 0.032 ± 0.016 |
| Lactates 1^st^ pneumonectomy | 0.027 ± 0.015 |
| Initial hemoglobin | 0.027 ± 0.014 |
| Body mass index of the donor | 0.027 ± 0.016 |
| Age mismatch | 0.026 ± 0.017 |
| Time on waiting list | 0.021 ± 0.010 |
| Preoperative pulmonary hypertension | 0.019 ± 0.018 |
| PNI score | 0.019 ± 0.011 |
| Lactates 2^nd^ graft implantation | 0.016 ± 0.010 |
| TLC missmatch | 0.016 ± 0.010 |
| Patent foramen ovale | 0.012 ± 0.009 |
| PaO2/FiO2 of the donor | 0.011 ± 0.007 |
| Initial lactates | 0.011 ± 0.007 |
| Age of the donor | 0.010 ± 0.007 |
| TLC of the donor | 0.010 ± 0.006 |

ECMO: extracorporeal membrane oxygenation ; PNI: prognostic nutritional score; TLC: Total lung capacity
