# Supplementary material for: Machine Learning for Predicting Pulmonary Graft Dysfunction After Double-Lung Transplantation: A Single-Center Study Using Donor, Recipient, and Intraoperative Variables
Source: Transpl Int. 2025 Oct 22;38:14965. doi: 10.3389/ti.2025.14965 (PMC12593525; doi:10.3389/ti.2025.14965)
Supplement: Supplementary file 7 [file Table3.docx]

**Supplementary Table 3.** Relative feature important in subgroup analysis 1: Only patients who never get ECMO (N=251)

| **Feature** |  |
| --- | --- |
| Lactate after anesthetic induction | 0.190 ± 0.061 |
| Lactates at 1^st^ pneumonectomy | 0.097 ± 0.043 |
| PaO_2_/FiO_2_ at 2^nd^ lung implantation | 0.061 ± 0.036 |
| Blood lactate level at 2^nd^ pneumonectomy | 0.061 ± 0.029 |
| Hemoglobin after anesthetic induction | 0.061 ± 0.026 |
| Lung Allocation Score | 0.056 ± 0.032 |
| 1^st^ lung ischemic time | 0.053 ± 0.029 |
| 2^nd^ lung ischemic time | 0.051 ± 0.030 |
| Age mismatch | 0.037 ± 0.023 |
| Lymphocytes | 0.037 ± 0.021 |
| Donor length of ventilation | 0.035 ± 0.020 |
| Blood lactate level at 1^st^ lung implantation | 0.029 ± 0.017 |
| Prognostic Nutritional Index score | 0.025 ± 0.016 |
| Body mass index | 0.023 ± 0.014 |
| Patent foramen ovale | 0.019 ± 0.013 |
| Donor chest Xray | 0.017 ± 0.014 |
| Age recipient | 0.016 ± 0.011 |
| TLC mismatch | 0.015 ± 0.010 |
| Donor PaO_2_/FiO_2_ | 0.014 ± 0.009 |
| TLC recipient | 0.014 ± 0.010 |

TLC: Total lung capacity
