# Supplementary material for: Machine Learning for Predicting Pulmonary Graft Dysfunction After Double-Lung Transplantation: A Single-Center Study Using Donor, Recipient, and Intraoperative Variables
Source: Transpl Int. 2025 Oct 22;38:14965. doi: 10.3389/ti.2025.14965 (PMC12593525; doi:10.3389/ti.2025.14965)
Supplement: Supplementary file 8 [file Table4.docx]

**Supplementary Table 4.** Relative feature important in subgroup analysis 2: Only patients who get ECMO (N=226)

| **Feature** |  |
| --- | --- |
| PaO2/FiO2 at 2^nd^ lung implantation | 0.162 ± 0.092 |
| TLC recipient | 0.126 ± 0.083 |
| TLC mismatch | 0.110 ± 0.076 |
| Age donor | 0.078 ± 0.071 |
| ECMO for hypoxia | 0.057 ± 0.053 |
| Blood lactate level at 2^nd^ pneumonectomy | 0.047 ± 0.056 |
| BMI donor | 0.046 ± 0.049 |
| BMI recipient | 0.045 ± 0.056 |
| Lymphocytes | 0.044 ± 0.051 |
| Date of transplantation | 0.041 ± 0.051 |
| Lung Allocation Score | 0.035 ± 0.047 |
| Bilirubin | 0.031 ± 0.040 |
| End-stage lung disease | 0.025 ± 0.035 |
| PaO2/FiO2 donor | 0.025 ± 0.031 |
| 1^st^ lung ischemic time | 0.024 ± 0.027 |
| Albumin | 0.018 ± 0.021 |
| Oto score | 0.018 ± 0.031 |
| Preoperative pulmonary hypertension | 0.014 ± 0.019 |
| Donor chest Xray | 0.014 ± 0.018 |
| Preoperative plasmapheresis | 0.014 ± 0.014 |

TLC: total lung capacity; ECMO: extracorporeal membrane oxygenation ; BMI: body mass index
